# Semicrystalline Polymer Micro/Nanostructures Formed by Droplet Evaporation of Aqueous Poly(ethylene oxide) Solutions: Effect of Solution Concentration

**DOI:** 10.1021/acs.langmuir.2c01872

**Published:** 2022-11-28

**Authors:** Shadi Kolahgar-Azari, Antonia Kagkoura, Dimitrios Mamalis, Jane R. Blackford, Prashant Valluri, Khellil Sefiane, Vasileios Koutsos

**Affiliations:** † School of Engineering, Institute for Materials and Processes, The University of Edinburgh, King’s Buildings, Edinburgh EH9 3FB, U.K.; ‡ Offshore Renewable Energy Catapult, Offshore House, Albert Street, Blyth NE24 1LZ, U.K.; § School of Engineering, Institute for Multiscale Thermofluids, The University of Edinburgh, King’s Buildings, Edinburgh EH9 3FD, U.K.

## Abstract

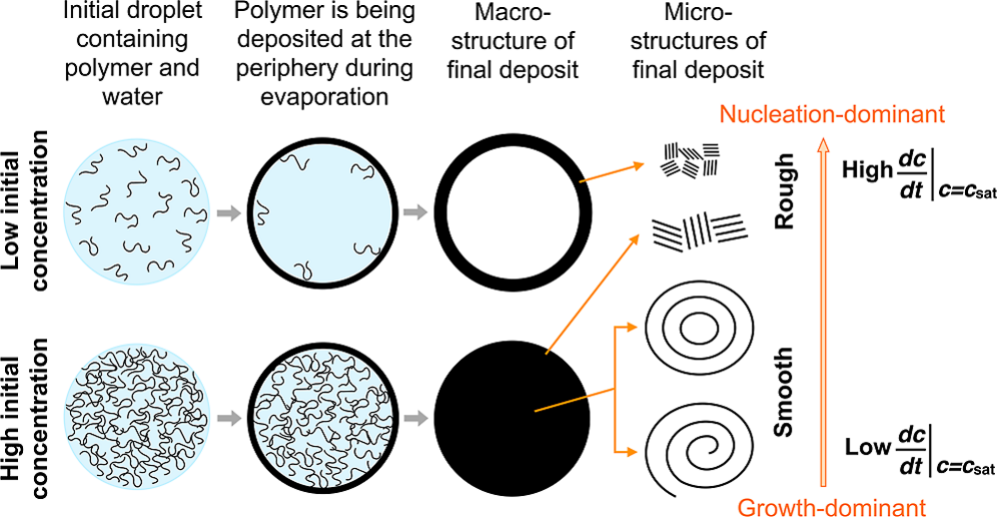

Deposits formed after evaporation of sessile droplets, containing aqueous solutions of poly(ethylene oxide), on hydrophilic glass substrates were studied experimentally and mathematically as a function of the initial solution concentration. The macrostructure and micro/nanostructures of deposits were studied using stereo microscopy and atomic force microscopy. A model, based on thin-film lubrication theory, was developed to evaluate the deposit macrostructure by estimating the droplet final height. Moreover, the model was extended to evaluate the micro/nanostructure of deposits by estimating the rate of supersaturation development in connection with the driving force of crystallization. Previous studies had only described the macrostructure of poly(ethylene oxide) deposits formed after droplet evaporation, whereas the focus of our study was the deposit micro/nanostructures. Our atomic force microscopy study showed that regions close to the deposit periphery were composed of predominantly semicrystalline micro/nanostructures in the form of out-of-plane lamellae, which require a high driving force of crystallization. However, deposit central areas presented semicrystalline micro/nanostructures in the form of in-plane terraces and spirals, which require a lower driving force of crystallization. Increasing the initial concentration of solutions led to an increase in the lengths and thicknesses of the out-of-plane lamellae at the deposits’ periphery and enhanced the tendency to form spirals in the central areas. Our numerical study suggested that the rate of supersaturation development and thus the driving force of crystallization increased from the center toward the periphery of droplets, and the supersaturation rate was lower for solutions with higher initial concentrations at each radius. Therefore, periphery areas of droplets with lower initial concentrations were suitable for the formation of micro/nanostructures which require higher driving forces, whereas central areas of droplets with higher initial concentration were desirable for the formation of micro/nanostructures which require lower driving forces. These numerical results were in good qualitative agreement with the experimental findings.

## Introduction

Tailoring the structure of deposits, formed by droplet evaporation, is beneficial in various industrial and biochemical applications.^1,2^ The coffee-ring effect,^3,4^ which causes the formation of a ringlike deposit upon evaporation of a droplet containing a suspension or solution, can be advantageous or disadvantageous depending on the application; the perimeter deposition of solutes after solvent evaporation is highly undesirable in inkjet printing and paint industries, whereas such directional deposition of solutes may be favorable for surface coating and patterning applications.^1,5,6^ The droplet evaporation process and the evaporation-induced deposition of solutes from the solution droplets have attracted a wide range of research interests from theory to practice in the subsequent years;^2^ however, a better understanding of the factors that contribute to the dynamics of the droplet evaporation and deposition is required to achieve effective approaches to tailor desired deposition patterns in both macro- and microscales. Such a study is more complicated for polymer solutions as the evaporation process of solutions containing macromolecules may involve the co-occurrence of interfacial interactions^7,8^ and phase transitions, for example, polymer crystallization,^9^ alongside heat and mass transfer. The evaporation process of droplets containing aqueous poly(ethylene oxide) (PEO) solutions and the unique patterns of the PEO deposits have recently aroused increasing research interest,^7,8,10−13^ and these studies have only focused on the structure of deposits in macroscales and not in micro/nanoscales.

PEO is a synthetic, highly water-soluble and biocompatible polymer which can readily be generated, processed, and modified. PEO-based materials are thus used in a wide range of biomedical applications^14^ including tissue engineering, implant surface modification, and drug-delivery systems. The linear molecules of PEO are crystallizable under thermodynamically and kinetically favorable conditions.^15−22^ Atomic force microscopy (AFM) is a powerful technique to investigate different micro/nanostructures of PEO.^20−25^ The objective of the present work was to experimentally and theoretically study the macrostructures and micro/nanostructures of the PEO deposits formed by droplet evaporation of aqueous PEO solutions with different initial concentrations.

The process of solute crystallization in a solution generally consists of three consecutive stages: solution supersaturation, nucleation, and growth of crystal. Evaporation of the solvent drives the solution to supersaturation, which refers to a state in which the solution contains more solute than can ordinarily dissolve at that temperature. Nucleation then occurs in a series of bimolecular collisions between solute molecules which form an aggregate of a small number of solute molecules. The clusters which exceed a critical size become stable nuclei. The solute molecules then diffuse from the surrounding solution to the surface of the nuclei and incorporate into the lattice, resulting in crystal growth. Crystallization of polymers involves the polymer chains folding back and forth, thereby forming crystalline regions called “lamellae.” The crystalline lamellae are separated by amorphous regions where there is no order in the arrangement of the polymer chains. Therefore, the degree of crystallinity in polymers never reaches 100%, and the polymer crystals are often called “semicrystalline.”^26−28^

The level of supersaturation is a critical parameter for solute crystallization in a solution, which is quantified in two ways: the supersaturation (Δ*c*) is defined as the difference between the supersaturated solution concentration (*c*) and its equilibrium or saturation concentration (*c*_sat_), and the ratio of supersaturation (*S*) is defined as the ratio of *c* to *c*_sat_.^29^The driving force of crystallization is the result of the difference in the chemical potential (Δμ) between a supersaturated solution and a solution which is in equilibrium, described as Δμ = *kT*ln *S*,^30^ where *k* is the Boltzmann constant, *T* is the temperature, and *S* is the ratio of supersaturation. The driving force of crystallization thus depends on the level of supersaturation.

**Figure 1:**
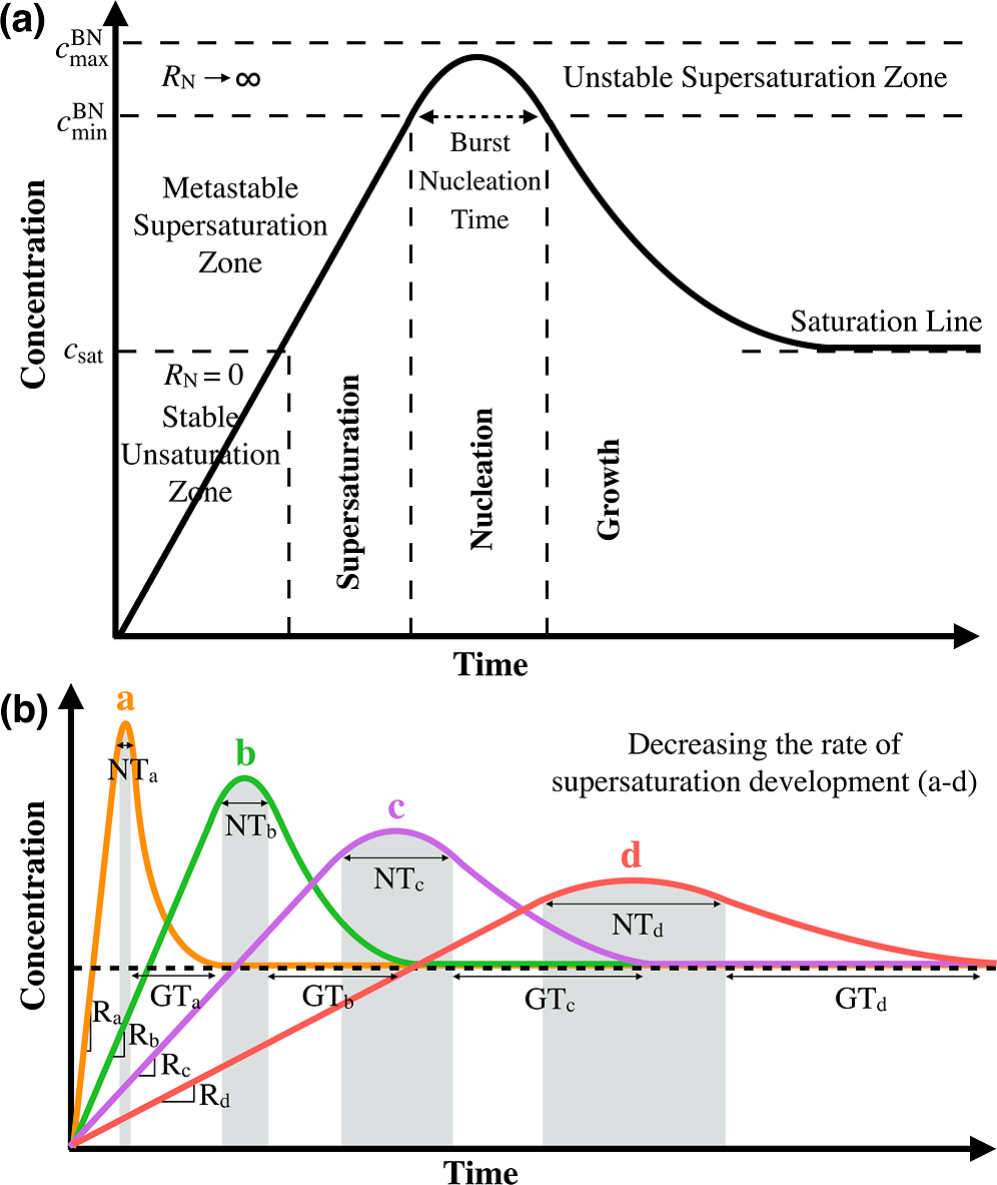
(a) LaMer diagram^35,36^ as a schematic representation of solute concentration with respect to time during three stages of solute crystallization in solution including solution supersaturation, nucleation, and growth of crystals. *c*_sat_ is the saturation concentration, whose difference with *c*_max_^BN^ and *c*_min_^BN^, respectively, constitutes the maximum achievable supersaturation and the minimum required supersaturation for burst nucleation. The nucleation rate (*R*_N_) is zero before supersaturation occurs and is infinite during the burst nucleation. (b) Schematic graph showing the effect of the rate of supersaturation development on the kinetics of crystal nucleation and growth; decreasing the rate of supersaturation development (*R*_a_ > *R*_b_ > *R*_c_ > *R*_d_) causes the nucleation time and the growth time to increase (NT_a_ < NT_b_ < NT_c_ < NT_d_ and GT_a_ < GT_b_ < GT_c_ < GT_d_).

The correlation between the solution supersaturation, homogenous nucleation, and growth of crystals is described by the LaMer mechanism of burst nucleation.^31−37^ The distinct stages of this mechanism are illustrated in the solubility profile shown in Figure 1a. A steady supply of solutes initially drives the solution from the stable unsaturated zone, where the nucleation rate is zero, into the metastable supersaturation zone, where the nucleation rate is low. The solution then goes into the unstable supersaturation zone, where the nucleation rate rises abruptly, and a large number of nuclei are simultaneously born. The growth of these nuclei causes the supersaturation to fall and thereby suppresses further nucleation. The growth continues until the concentration reaches saturation concentration, where an equilibrium state is reached. The kinetics of nucleation significantly depends on the initial supersaturation or the rate of supersaturation development,^38−41^ that is, the rate of change in concentration over time during supersaturation development. The rate can be either constant^38^ or variable^39^ before the burst nucleation depending on the experiment, but is always continuous. In either case, the rate of supersaturation development strongly affects the minimum supersaturation required for burst nucleation, the maximum achievable supersaturation, the time when the burst nucleation starts, and the length of time when nucleation and the following growth lasts, as shown in Figure 1b. A high rate of supersaturation development leads to a surge in the minimum supersaturation required for burst nucleation and the maximum achievable supersaturation. It is shortly followed by the nucleation burst, which occurs in a short period of time, and then a rapid decrease in solute concentration during which growth is completed. However, a slow rate of supersaturation development causes a lag time to appear before the formation of any detectable nuclei. The rate of the following nucleation and growth is also low, which results in a lower but broader solubility profile. Depending on the kinetics of crystallization, either nucleation or growth may be predominant, leading to the formation of crystals with different structures^30,42^ and sizes.^29^

### Background on Experimental Studies

Experimental studies have shown that the macrostructure of the PEO deposits varies from a ringlike to puddlelike pattern, with or without a central pillar,^7,8,10−12^ depending on the initial polymer concentration in solution,^8,10,11^ the weight-average polymer molecular weight,^7,8^ and the evaporation rate which is controlled by the ambient conditions, that is, temperature, humidity, and pressure,^11,12^ or through increasing the heating rate of the substrate.^13^ All these studies have only focused on the macrostructure of the PEO deposits, which can be observed by the naked eye or at very low levels of magnification, that is, below 10×. However, the effect of these parameters on the micro/nanostructures of the PEO deposits is still unknown, which can only be revealed by the microscopy techniques under high levels of magnification. This knowledge is essential to accomplish a more controlled evaporation-induced deposition mechanism.

### Background on Model-Based Studies

Models based on thin-film lubrication theory^43−48^ to describe the macrostructure of deposits have shown that deposits are influenced by the interplay between various evaporation-induced phenomena, mainly including solvent evaporation, radial convection of fluid, and solute advection caused by the coffee-ring effect. The effect of gravity has been ignored in these studies, considering the small value of Bond number, , in which ρ_0_ is the liquid density, *g* is the gravity acceleration, *R* is the droplet radius, and γ_0_ is the interfacial tension. The insignificant Bo number ensures the negligible effect of gravity on the droplet interface compared to the effect of surface tension. The effects of Marangoni flow and vertical solute diffusion have been ignored too, considering the small ratio of vertical to horizontal length scale of the thin droplets. The effect of horizontal solute diffusion^47^ has been characterized by the Peclet number, , which estimates the relative importance of advection versus diffusion of solutes.^48^ Here, *h*_0_ is the initial height of the droplet, *j* is the evaporation flux, and *D* is the diffusion coefficient. These models have been used to calculate the time evolution of the droplet height and/or the solute mass distribution in the droplet during evaporation. The resulting deposits have been shown to have a ringlike or puddlelike structure under different conditions. In particular, the initial solution concentration plays a pivotal role in predicting the macrostructure of the deposit.^44−46,48^ However, the studies have not provided an understanding of the key drivers affecting the micro/nanostructures of the deposit.

### Current Study and Contributions

The present work experimentally and mathematically studied the effect of initial solution concentration on the macrostructure and micro/nanostructures of the PEO deposits left after droplet evaporation. In summary, the Experimental Section describes the droplets containing the PEO solutions with different initial concentrations that were left to evaporate on hydrophilic glass substrates, and then the macrostructure and micro/nanostructures of the resulting deposits were studied by stereo microscopy and AFM, respectively. Then, the Theoretical Modeling Section describes the model which was developed, based on thin-film lubrication theory, to predict the macrostructure of deposits by calculating the final height of the droplets, . The model was then extended to also predict the micro/nanostructures formed in different regions of the deposits by calculating the solute concentration for all radii, *c*(*r*,*t*), and thus the concentration change per unit time at the saturation point, d*c*/d*t*|_*c*=*c*_sat__, for all radii. d*c*/d*t*|_*c*=*c*_sat__ constitutes the value of the derivative calculated at the time when the concentration equals its saturation level. The value of d*c*/d*t*|_*c*=*c*_sat__ at each radius gives an estimate for the rate of supersaturation development at that radius, which affects the driving force of crystallization and thus the micro/nanostructures formed at that area of the deposit. By comparing the values of d*c*/d*t*|_*c*=*c*_sat__ around different regions examined by AFM, the model qualitatively justifies the formation of relevant polymer micro/nanostructures at each area.

## Experimental Section

Aqueous PEO solutions with the fixed molecular weight of 100 kg/mol (Sigma-Aldrich, 44101) and four different initial concentrations of 0.01, 0.1, 1 and 10% (w/w) were prepared. To prepare the solutions, PEO powders and distilled, deionized water were mixed and left to equilibrate for about 24 h. Meanwhile, a roller mixer was employed in the solution to increase the dissolution rate and eliminate the sedimentation effect. To remove the probable undissolved clusters of the polymer chains, the solutions were filtered through 0.45 μm pore size filters (by Sigma-Aldrich) using a syringe pump assembly with adjustable speed. The filtering speed was kept low and constant at 0.5 mL per hour in order not to affect the polymer molecules. Filtering the solutions results in only a slight reduction of overall solution concentrations,^7^ which is ignored. A 1 mL syringe with a metallic syringe needle of 0.64 mm diameter (by Sigma-Aldrich) was used to place the droplet with the initial volume of 3 μL on the substrate. The solid substrate used in this study was borosilicate glass microscope coverslip (by TAAB Laboratories Equipment Ltd, England). The glass substrate was hydrophilic with a low initial contact angle (<45°) and had a circular shape with dimensions of 10 mm diameter and 0.085–0.13 mm thickness. Before use, the glass substrates were cleaned with ethanol and acetone. A spirit level was used to ensure that the substrates were horizontal. The droplets’ evaporation was then performed under controlled environmental conditions: relative humidity of 36 ± 5%, temperature of 18 ± 1 °C, and pressure of 1 atm.

To analyze the polymer deposits after the droplet evaporation and deposition on the substrate, a stereo microscope (Zeiss Stemi 2000, Germany) was used to display the resulting patterns in the macroscale. The images were then analyzed using Zeiss AxioVision 4.9.1 software. A digital camera and a diffuse light source, placed on either side of the deposits, were also used to take the side-view image of the deposits. To study the surface microstructures of the polymer deposits, an atomic force microscope (AFM Multimode/Nanoscope IIIa, Bruker, USA) equipped with a J-scanner (*x*–*y* range ≈ 140 μm) was used. The glass substrate upholding the deposit was fastened to a magnetic disc with a double-sided adhesive tape, and the disc was placed on the AFM sample holder. The AFM study was performed in the tapping mode in the ambient condition at the relative humidity of 35 ± 5%. The AFM probe used in this study had a symmetric rotated tip with the radius of 8–12 nm (model MPP-11220-10, Bruker). The cantilever was 0.01–0.025 Ω cm antimony (*n*)-doped Si with reflective aluminum coating on the backside, with a nominal spring constant of 40 N/m and a resonance frequency of 300 kHz. To minimize the interactions between the tip and the sample during imaging, the set-point amplitude ratio was kept not less than 0.5. The images were then processed and analyzed using Gwyddion software.^49^

## Theoretical Modeling

### Problem Definition and Assumptions

**Figure 2:**
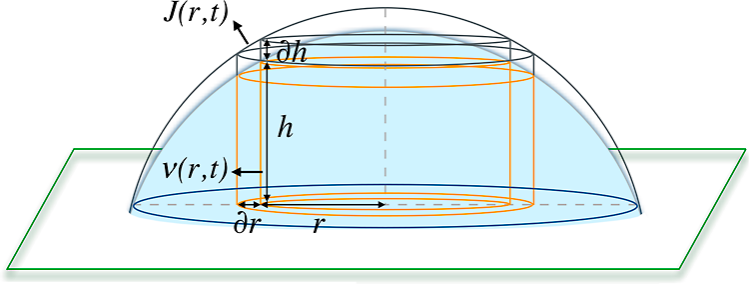
Schematic diagram of a pinned droplet evaporating based on the coffee-ring model. An annular cylinder section is considered within the droplets, whose geometric parameters include the radial distance from the center *r*, height *h*, infinitesimal wall thickness ∂*r*, and infinitesimal height change ∂*h*. ν(*r*,*t*) and *J*(*r*,*t*), respectively, represent the velocity of radial outward fluid flow and the volumetric evaporation flux.

We considered a pinned evaporating droplet placed on a flat solid substrate as shown in Figure 2. Similar to the coffee-ring model,^3,4^ the droplet was assumed to contain a nonvolatile solute of density ρ_s_ dissolved in a volatile solvent of density ρ. An annular cylindrical section of infinitesimal thickness ∂*r* and evaporation-induced height reduction ∂*h* was considered within the droplet, with the radial distance *r* from the center of the droplet and the height *h*(*r*,*t*). The velocity of fluid flow, ν(*r*,*t*), which was considered to be uniform in this section, corresponds to the vertically averaged volume of fluid flowing in the radial outward direction per unit of area per unit of time. The volumetric evaporation flux, *J*(*r*,*t*), constitutes the volume of fluid evaporating per unit surface area of droplet per unit of time.

The droplets were very thin, which enabled us to use the lubrication approximation,^43−48^ such that the flow in the vertical and circumferential directions was noticeably smaller than the radial direction. Based on our experiments, the overall aspect ratio of droplets, defined as , was small, where *h*_0_ is the initial height of the droplet in the center (∼0.4 mm) and *d*_0_ is the initial droplet diameter (∼3 mm). Given the height of our droplets was smaller than the expected capillary length, we ignored gravity. We also ignored Marangoni flow and vertical solute diffusion within the thin droplets.^43−48^ Our experimental Peclet numbers were high, , indicating that the advection mass transfer dominated the diffusion mass transfer.^11,12,44,48^ Thus, the horizontal diffusion of solutes was also ignored.

### Mass Conservation in the Fluid

The total mass change in the annular section, shown in Figure 2, due to the radial outflow of fluid and evaporation is
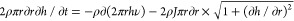
1where ρ is the density of the fluid. The effect of curvature in the annular region, ∂*h*/∂*r*, is small and can be neglected.^4^ It is due to its geometry in a thin droplet which becomes smaller by squaring, and thus its magnitude is much smaller compared to the value “1” in the formula. The mass balance thus reduces to
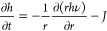
2

The solute concentration, *c*(*r*,*t*), increases as evaporation proceeds and eventually exceeds a specific value, namely, the saturation concentration, *c*_sat_, where the solute crystallization starts. The evaporation ceases where crystallization occurs. The evaporation flux thus vanishes as soon as the solute concentration reaches the value where the transition of the system to the solid state occurs.^44,45,47^ Moreover, the evaporation flux varies with radial distance and time throughout the droplet profile, *h*, and has the highest value at the triple contact line where *h* = 0.^43,45−47,50^ Considering the effects of the solute concentration increase^44,45,47^ and the droplet height change^43,45−47,50^ during evaporation, the evaporation flux, *J*, is described as follows
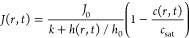
3where *J*_0_ is the evaporation flux of the pure solvent, which is assumed to be constant and independent of the droplet shape, and *k* is a dimensionless parameter representing the relative volatility. *k* → ∞ for nonvolatile substances and *k* → 0 for high volatile substances, while *k* was here set to 1, based on an exhaustive search around the *k* values reported in.^43,45−47,50^

### Mass Conservation for Solute

The solute concentration varies at any radial distance during the solvent evaporation time. Similarly, the total mass change of the solute in the annular section as a result of the radial outward fluid flow gives,^4^
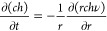
4

### Radial Velocity of Fluid

The actual velocity of fluid flow at radial distance *r*, height *z*, and time *t*, ν(*r*,*z*,*t*), was obtained through Navier–Stokes equations, simplified by the lubrication (thin-film) approximation suitable for thin droplets^43−48^ given by
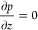
5
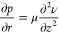
6where *p* and μ are, respectively, the pressure and viscosity of the fluid. ν(*r*,*z*,*t*) was obtained using two boundary conditions of no shear stress, ∂ν/∂*z* = 0, at the liquid–air interface where *z* = *h*(*r*,*t*), and no slip of droplet, ν = 0, on the substrate where *z* = 0. The height-average velocity, ν(*r*,*t*), was thus obtained as
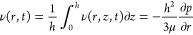
7

Here, *p*(*r*,*t*) was derived from the Young–Laplace equation, which describes the pressure difference across the interface between the fluid and the atmosphere due to the phenomenon of interfacial tension,
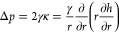
8in which γ is the interfacial tension and κ is the interface mean curvature of the thin film.^44−46^ Thus
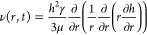
9

Here, μ is the viscosity of the fluid which increases during evaporation due to the increase in solute concentration, given by^46^
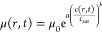
10where μ_0_ is the viscosity of the pure solvent and *a* and *b* constants, representing how quickly μ increases when *c* tends to *c*_sat_, were set to 10 and 8, respectively. These values were estimated based on an exhaustive search around the values reported in.^46^

### Boundary and Initial Conditions

We employed the following boundary conditions at the droplet periphery and the droplet center.
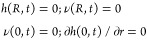
11where *R* is the initial droplet contact radius. The initial droplet was considered to be in the form of a sphere cap, with the height described as
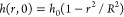
12

The initial concentration of the solute was considered constant throughout the droplet as
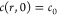
13

The calculations were carried out for four initial concentrations, *c*_0_, of 0.01, 0.1, 1, and 10%. The formation of semicrystalline PEO occurs where the polymer concentration is sufficiently high. It corresponds to the local saturation concentration, which remains roughly constant at *c*_sat_ ≈ 0.6 irrespective of the PEO molecular weight.^7,8^ The initial height of the droplet in the center and the initial droplet radius were *h*_0_ = 0.4 mm and *R* = 1.5 mm, based on the experiment. The evaporation flux and viscosity of water are *J*_0_ = 24 × 10^–4^ m/s and μ_0_ = 8.9 × 10^–4^ Pa s in our experimental setting.

### Numerical Solution

Eqs 2, 4, and 9 were numerically solved using finite difference methods subject to the boundary and initial conditions. For that, the differential equations were discretized with the time step Δ*t* = 10^–3^ and the normalized space step Δ*r* = 10^–2^. The difference equations were represented by the sparse matrix equations, which were then solved iteratively in Matlab software to obtain *h*(*r*,*t*) and *c*(*r*,*t*), as described in the Supporting Information. Accordingly, the final height, , and the concentration changes per unit of time on the saturation boundary, d*c*/d*t*|_*c*=*c*_sat__, for different droplet radii were calculated.

## Results and Discussion

### Experimental Results

**Figure 3:**
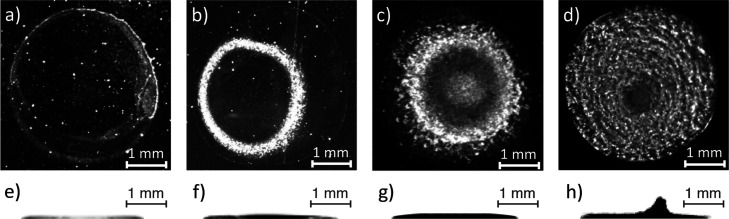
Top-view stereo microscope images (a–d) and respective side-view images (e–h) of the deposits formed after the droplet evaporation of 100 kg/mol PEO solutions with the initial concentrations of 0.01, 0.1, 1, and 10% (w/w), respectively.

The polymer deposits remained on the glass substrate upon evaporation of sessile droplets containing 100 kg/mol PEO solutions with the initial concentrations of 0.01, 0.1, 1, and 10% (w/w), as shown in Figure 3. The figure presents the top-view stereo microscope images and the side-view digital camera images of the deposits. Based on the images, the macrostructure of the deposits changed from a ring to a tall central pillar formed over a disklike puddle, as the initial concentration of solutions increased from 0.01 to 10% (w/w). At 0.01% solution, a single ring was formed, while at 0.1% solution, two semiconcentric rings were formed owing to the stick-slip behavior^51,52^ of the droplet contact line during evaporation. The 1% solution deposit formed a central puddle and also a ring, as shown in the top-view image that reveals the accumulation of material at the periphery in addition to the central area. The formation of the distinctive tall conical pillar observed in the center of 10% solution deposit is consistent with the results of previous studies on pillar formation in pinned evaporating droplets of PEO solutions with different initial concentrations^8,10,11^ and molecular weights of the polymer.^7,8^ In such studies, the droplet evaporation of 100 kg/mol PEO solution with the initial concentration of 10% on hydrophilic substrates has often been associated with pillar formation, as observed in the present study too. Furthermore, based on the side-view images, the maximum height of deposits was around 0.1 mm, except for the pillar part of 10% solution deposit which had the maximum height of about 0.5 mm. The radius of deposits was also evaluated as about 1.5 mm.

**Figure 4:**
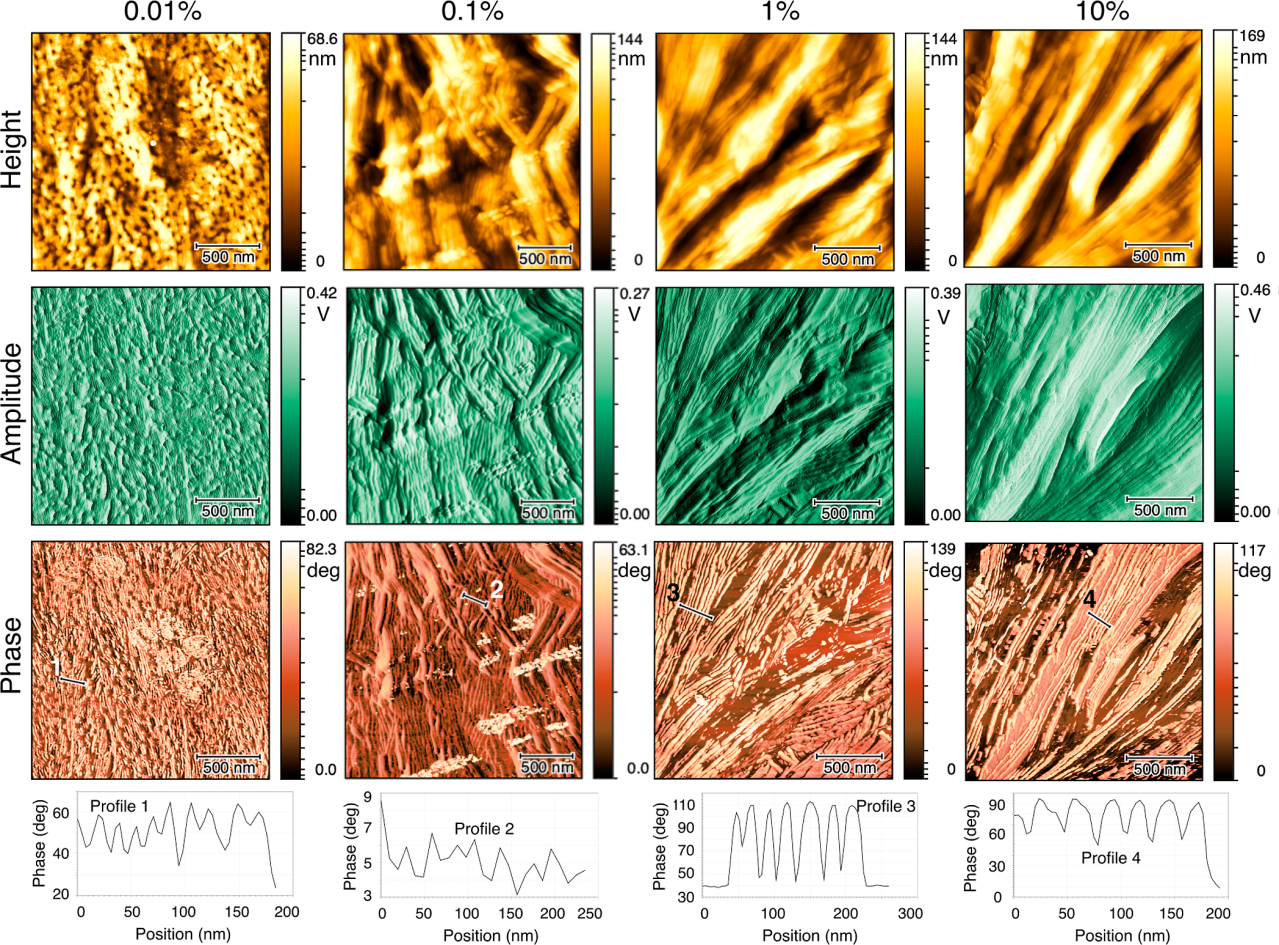
Height, amplitude, and phase AFM images taken from the periphery areas of the PEO deposits with the initial concentrations of 0.01, 0.1, 1, and 10% (w/w). The phase profiles of the corresponding lines are presented in the bottom row.

**Figure 5:**
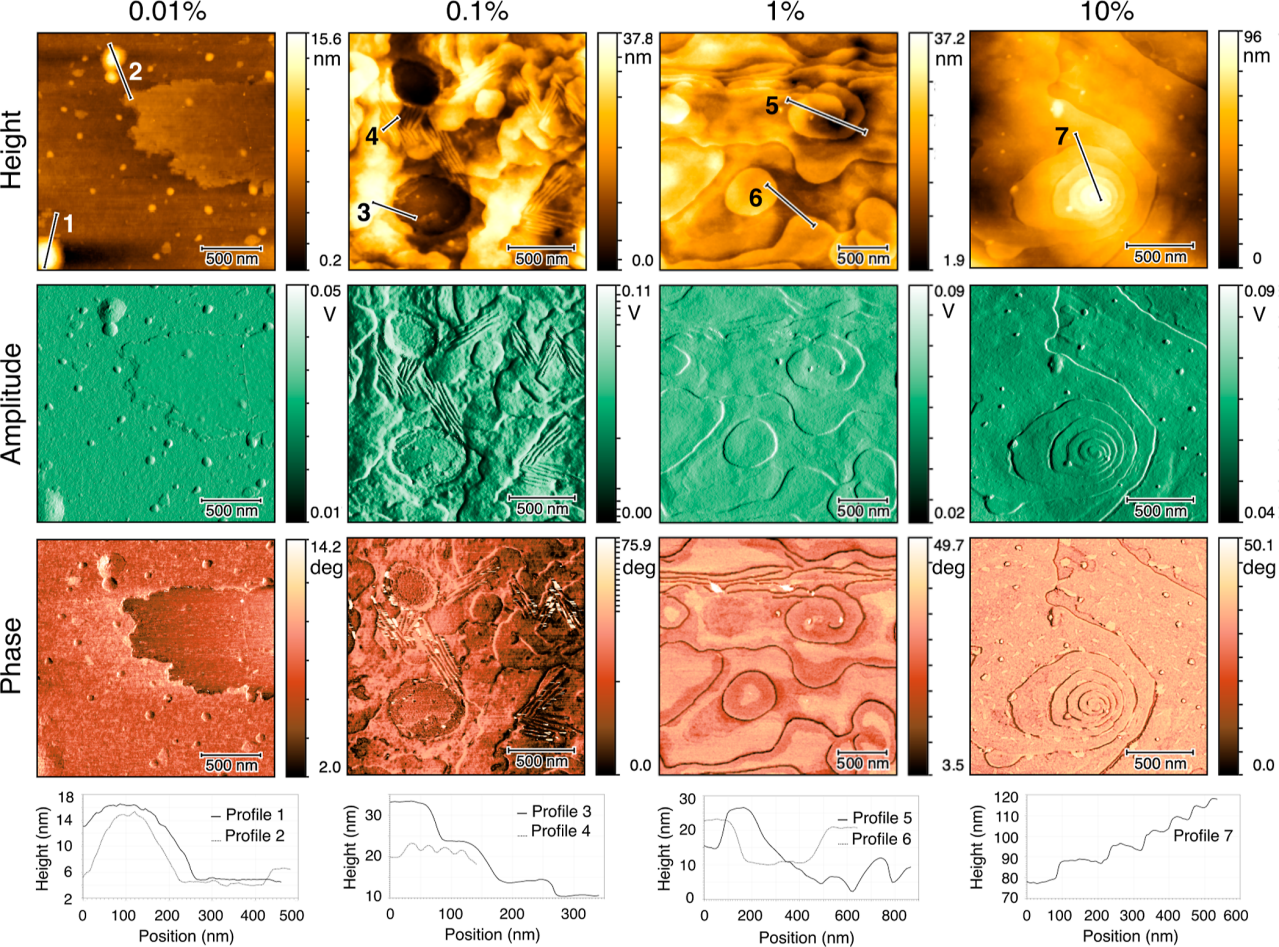
Height, amplitude, and phase AFM images taken from the central areas of the PEO deposits with the initial concentrations of 0.01, 0.1, 1, and 10% (w/w). The height profiles of the corresponding lines are presented in the bottom row.

**Figure 6:**
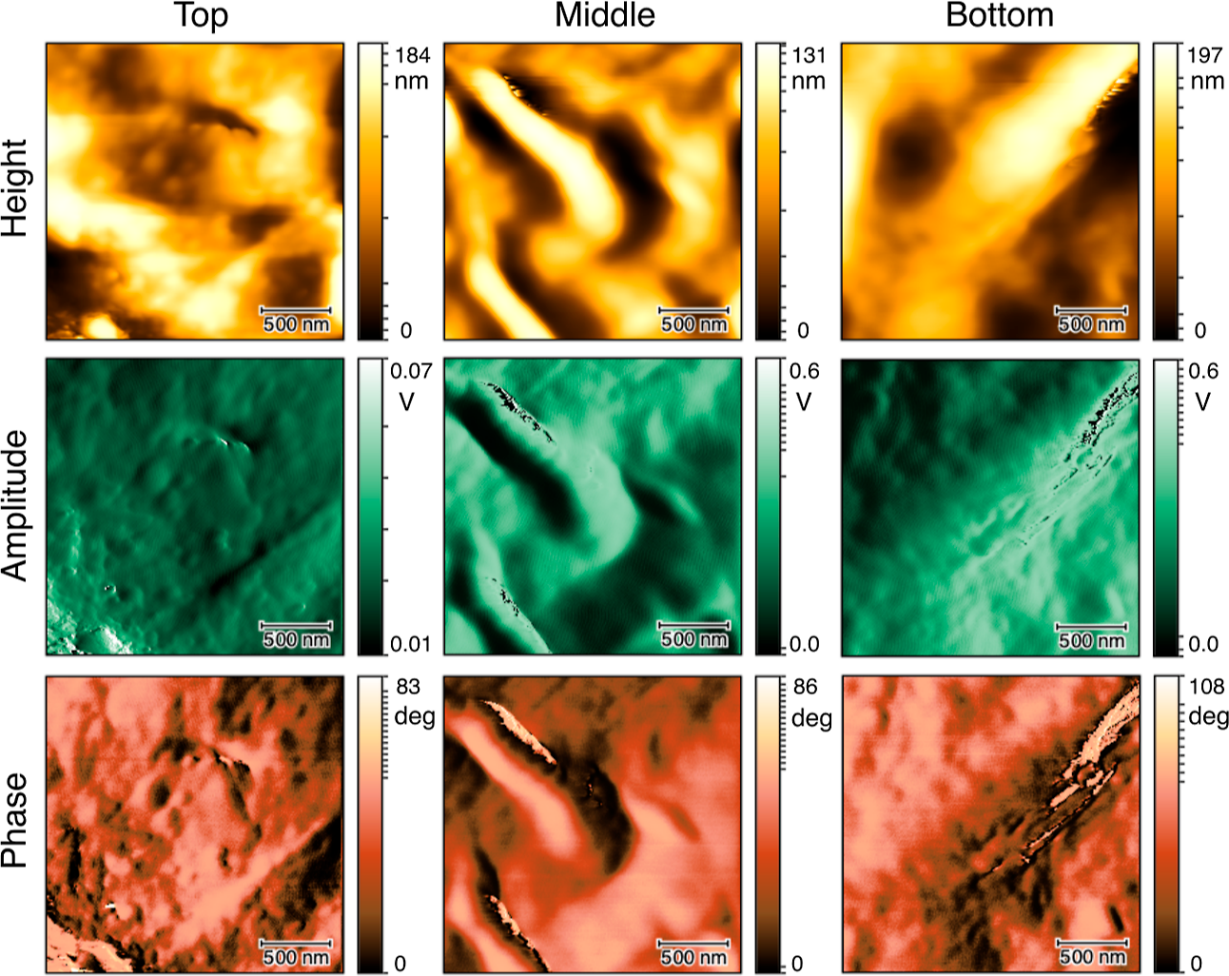
Height, amplitude, and phase AFM images taken from the top, middle, and bottom of the pillar formed in the center of the PEO deposit with the initial concentration of 10% (w/w).

The polymer micro/nanostructures, observed by AFM, over the periphery and central areas of the deposits are shown in Figures 4 and 5, respectively. By “periphery,” we mean the adjacent area of the deposit boundary, and by “central,” we mean the area from the center of the deposit toward the periphery excluding the pillar. From left to right, the figures represent the results for the 100 kg/mol PEO solutions with the initial concentrations of 0.01, 0.1, 1, and 10% (w/w). The images in the first, second, and third rows constitute the height, amplitude, and phase AFM images, respectively. The phase profiles or height profiles of the corresponding lines, indicated on the phase or height AFM images, are shown in the last rows. Besides, the polymer micro/nanostructures formed over the top, middle, and bottom of the pillar, located in the center of the 10% solution deposit, are shown in Figure 6.

The micro/nanostructures formed in the periphery areas of the deposits are presented in Figure 4. The periphery areas of all deposits were observed to contain semicrystalline micro/nanostructures in the form of out-of-plane lamellae (see Figure 4). The geometric dimensions of the lamellae varied with the initial concentration of solutions, which are described quantitatively later in this section.

The micro/nanostructures formed in the central areas of the deposits, excluding the pillar, are shown in Figure 5. In the central areas of the deposits, the stacks of out-of-plane lamellae were not observed regularly (see Figure 5). The 0.01% solution deposit contained few amorphous micro/nanostructures in the form of distinct small droplets adsorbed on the substrate^53^ in the central area of the deposit (see Figure 5, the first column). The central area of 0.1% solution deposit consisted of in-plane terraces and infrequent stacks of out-of-plane lamellae (see Figure 5, the second column). The terrace structures, which were made of flat-lying or nearly flat-lying layers located on top of each other, were characterized by a stair-step topography. The step height of the first layer (∼5 nm), located directly over the substrate, was lower than the step heights of other layers on top of the first layer (∼10 nm) (see Figure 5, the second column). The deposits of 1 and 10% solutions were mainly made of in-plane terraces and spirals in the central areas (see Figure 5, the third and fourth columns). The spiral structures were characterized by a curved layer revolving around a fixed center of rotation at an increasing distance from the base plane. The layer height of the spiral, shown in the fourth column of Figure 5, was observed to gradually decrease from about 9 to 5 nm, moving from the base plane toward the vertex. As the initial concentration of the solutions was increased from 0.1 to 10%, the chance of out-of-plane lamellae formation was observed to decrease, while the chance of spiral formation was increased in the central areas of the deposits (see Figure 5).

The micro/nanostructures formed over the conical pillar, in the center of the PEO deposit with the initial concentration of 10% (w/w), are shown in Figure 6. The AFM images were taken from the top, middle, and bottom areas of the pillar. In the center of the 10% solution deposit over the conical pillar, the semicrystalline structures were rarely found, and the polymer structures were predominantly amorphous (see Figure 6).

**Figure 7:**
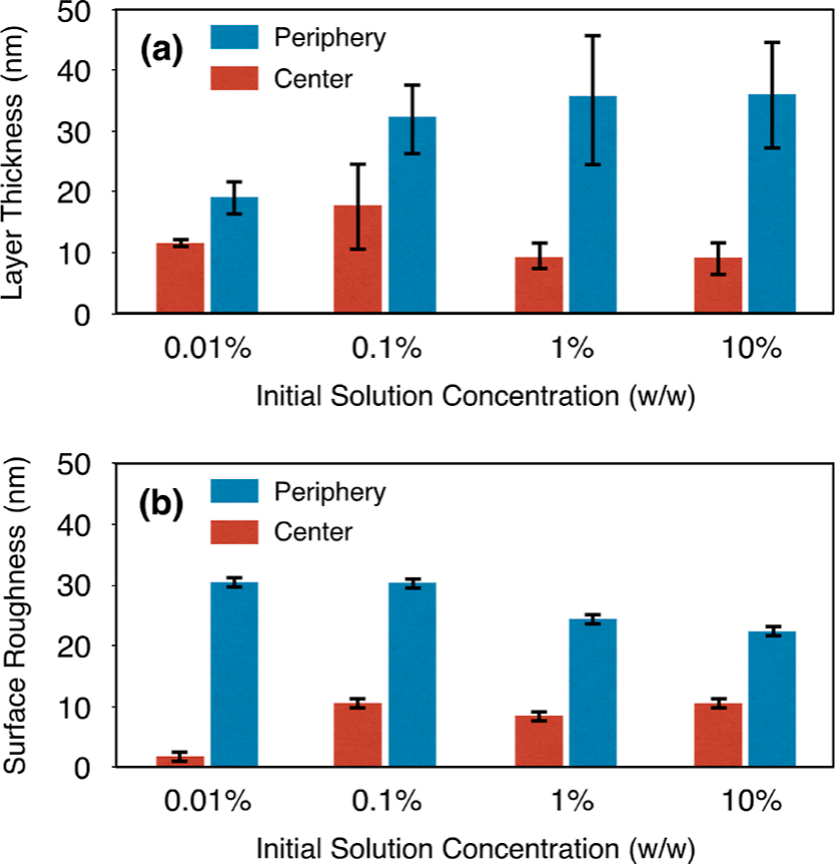
Bar charts illustrating the (a) layer thickness and (b) surface roughness, with error bars, in the periphery areas (blue bars) and central areas (red bars) of the PEO deposits with the initial concentrations of 0.01, 0.1, 1, and 10% (w/w). The layer thickness data are generated by cross-cutting the AFM images and measuring the valley-to-valley intervals for out-of-plane lamellae and the step heights for in-plane terraces and spirals. The surface roughness is determined by measuring the rms surface roughness.

The data collected from the profiles of various cross-cuts in the AFM phase and height images were analyzed, and the results are shown as bar charts with error bars in Figure 7a. The data were collected through measuring the valley-to-valley intervals of the phase profiles for the out-of-plane lamellae (see Figure 4) and measuring the step heights for the in-plane terraces and spirals (see Figure 5). The phase images gave a better view of the out-of-plane lamellae compared with the height images, which was the reason why the phase images were used to measure these layer thicknesses. Figure 7b shows the result of measuring the root-mean-square (rms) surface roughness for each AFM height image. The bar charts summarize the analysis results for the periphery (blue bars) and central areas (red bars) of the deposits with the initial concentrations of 0.01, 0.1, 1, and 10% (w/w).

The geometric dimensions of the layers, that is, thickness and length, varied for different initial concentration solutions in the periphery areas of the deposits (see Figure 4). The length of the lamellae was lower for the deposits of solutions with lower initial concentrations, for example, below 100 nm for 0.01% solution, than for the deposits of higher initial concentration solutions, for example, above 1 μm for 10% solution (see Figure 4). Similarly, the average thickness of the layers was lower for the deposits’ periphery of solutions with lower initial concentrations, for example, 19 nm for 0.01% solution, and the average thickness increased to 36 nm as the initial concentration was increased to 10% (see Figure 7a, for periphery). However, in the central areas, where out-of-plane lamellae were infrequently found (see Figure 5), the average thickness of layers was always below 18 nm (see Figure 7a, for center). Besides, the surface roughness was considerably high in the periphery areas (see Figure 7b), where large quantities of out-of-plane lamellae were found (see Figure 4), compared to the central areas where such rough structures were infrequently found (see Figure 5). The surface roughness of the periphery area had a downward trend as the initial concentration of solutions was increased (see Figure 7b) in association with increasing the layer thickness (see Figure 7a) and length (see Figure 4).

### Numerical Results

**Figure 8:**
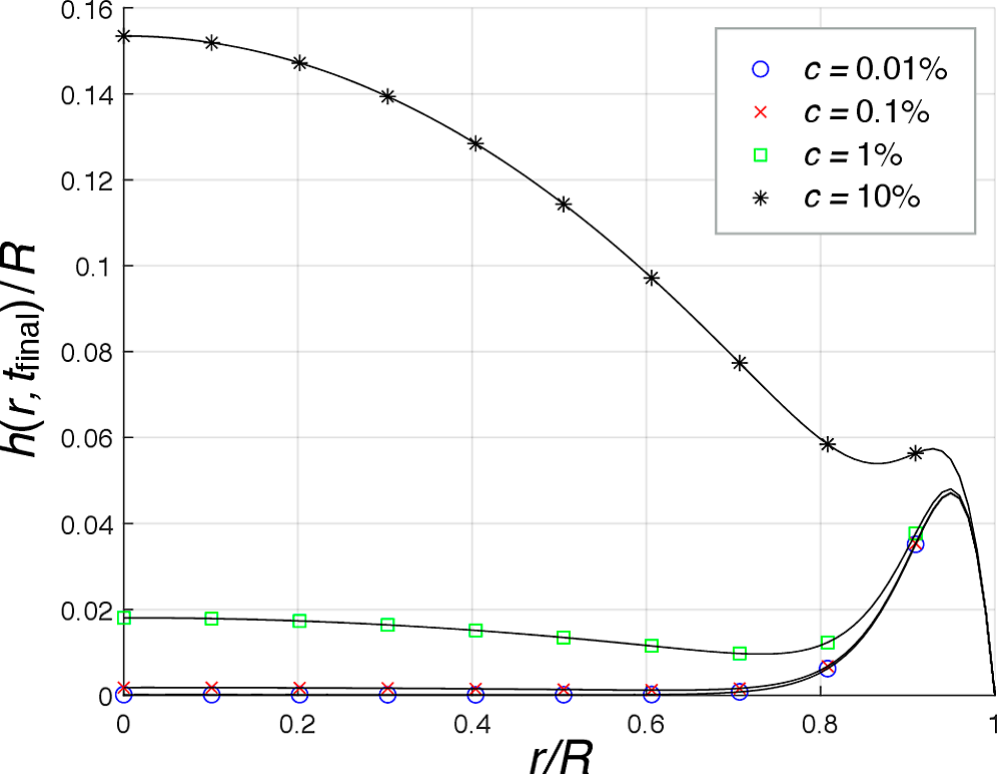
Normalized final height of droplets showing the macrostructure of deposits;  with respect to *r*/*R* for four solution droplets with the initial concentrations of 0.01, 0.1, 1, and 10% (w/w).

The numerical results of the final height calculations, which were carried out for four solution droplets with the initial concentrations of 0.01, 0.1, 1, and 10% (w/w), are shown in Figure 8. The figure illustrates the normalized profile of final height, , with respect to the normalized radius, *r*/*R*, for each droplet. Accordingly, the deposit of 0.01% solution had a ringlike macrostructure as the deposited material was accumulated in the periphery area. The deposit of 0.1% solution also had a similar ringlike macrostructure, whereas some more amount of material was deposited in the central area too. The maximum value of  was about 0.047 for both deposits of 0.01 and 0.1% solutions close to their peripheries. The level of  was slightly higher in the central area of 0.1% deposit, that is, about 0.002, compared to the central area of 0.01% deposit where  was nearly zero. The formation of the second ring in 0.1% deposit observed in the experiment (see Figure 3b) was not demonstrated in the numerical study. The deposit of 1% solution had similarities to both a ring and a well-formed puddle according to the numerical result as a noticeable amount of material was shown to deposit not only in the periphery but also in the central area. The 1% deposit had higher values of  in both the periphery and central areas compared to 0.01 and 0.1% deposits, with the maximum  of about 0.048 in the periphery area and  of about 0.018 in the center of deposit. The deposit of 10% solution had a puddlelike macrostructure according to the numerical result, in which the formation of a central pillar observed in the experiment (see Figure 3d,h) was not demonstrated. The 10% deposit had the highest values of  in all radii compared to other deposits, with the maximum  of about 0.154 in the center.

**Figure 9:**
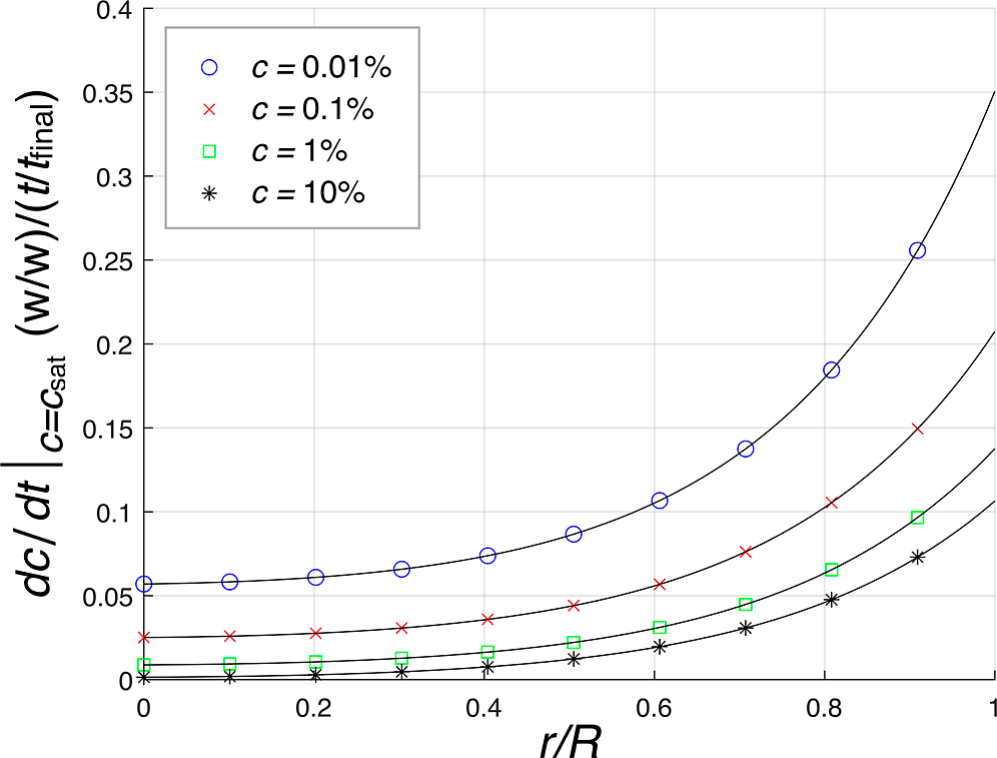
Concentration change per unit of time on the saturation boundary, d*c*/d*t*|_*c*=*c*_sat_,_ with respect to *r*/*R* for four solution droplets with the initial concentrations of 0.01, 0.1, 1, and 10% (w/w).

The numerical results obtained from calculating the concentration change per unit of time on the saturation boundary, d*c*/d*t*|_*c*=*c*_sat__, at each normalized radius, *r*/*R*, for the droplets containing the initial solution concentrations of 0.01, 0.1, 1, and 10% (w/w) are given in Figure 9. The value of d*c*/d*t*|_*c*=*c*_sat__ at each radius gives an estimate for the rate of supersaturation development at that radius, which affects the micro/nanostructures formed at that area of the deposit. According to the numerical results, the profiles of d*c*/d*t*|_*c*=*c*_sat__ followed an upward trend from the center to periphery of droplets, with a further increase of the slope on getting closer to the periphery. The level of d*c*/d*t*|_*c*=*c*_sat__ for 0.01% solution was thoroughly over 0.05, which increased from 0.058 in the center to 0.35 at the periphery. The 0.1% solution had the minimum d*c*/d*t*|_*c*=*c*_sat__ of 0.025 in the center, which increased to 0.21 toward the periphery. For 1% solution, the minimum and maximum values of d*c*/d*t*|_*c*=*c*_sat__ were about 0.01 in the center and about 0.14 at the periphery, respectively. For 10% solution, the level of d*c*/d*t*|_*c*=*c*_sat__ increased from nearly zero in the center to about 0.11 at the periphery. Thus, the central areas of all droplets had the lowest levels of d*c*/d*t*|_*c*=*c*_sat__, while the periphery areas had the highest levels of d*c*/d*t*|_*c*=*c*_sat__ in each droplet. Moreover, the solutions with higher initial concentrations had lower levels of d*c*/d*t*|_*c*=*c*_sat__ in each radius and smaller changes of d*c*/d*t*|_*c*=*c*_sat__ from the center to periphery compared to the solutions with lower initial concentrations. The levels of d*c*/d*t*|_*c*=*c*_sat__ were thus higher for the solutions with lower initial concentrations in each radius, and the slopes of profiles were noticeably steeper. Besides, the value of d*c*/d*t*|_*c*=*c*_sat__ nearly approached zero in the center of 10% solution droplet, where the pillar formation was shown to occur in the experiment (see Figure 3d,h). The levels of d*c*/d*t*|_*c*=*c*_sat__ in all four solutions were over 0.09 near the periphery areas where *r* > 0.95*R*, while the levels of d*c*/d*t*|_*c*=*c*_sat__ were lower, that is, below 0.09, within the central areas where *r* < 0.5*R*.

### Discussion and Interpretation of Findings

The different micro/nanostructures over the PEO deposits were investigated and categorized based on the AFM analysis results. The AFM study revealed that the micro/nanostructures over the PEO deposits, which remained on the glass substrate upon evaporation of droplets containing 100 kg/mol PEO solutions with the initial concentrations of 0.01, 0.1, 1, and 10% (w/w), can be divided into two main categories: semicrystalline structures and amorphous structures. The semicrystalline structures, which exhibit highly ordered hierarchical arrangements of alternating crystalline and amorphous layers, were observed to be in the form of out-of-plane lamellae (see Figure 4), in-plane terraces, and spirals (see Figure 5).

The AFM images of the micro/nanostructures formed in the periphery areas of deposits were investigated. The randomly oriented stacks of out-of-plane lamellae were the only form of semicrystalline structures observed in the periphery areas of the deposits (see Figure 4). The stacks of out-of-plane lamellae were possibly parts of frustrated spherulites. A spherulite^26−28^ is a polycrystalline aggregate of alternate crystalline and amorphous layers, which is formed by consecutive branching and reorientation of the lamellae in noncrystallographic directions. The reorientations are related to the thermal and mechanical fields and/or concentration gradients which are built around the crystallites during growth. The most common mechanism of spherulite growth is described as the small-angle branching,^54^ where the successive branches experience a limited liberation from the directions imposed by the crystal symmetry. The crystalline lamellae with the gaps of the amorphous layers grow with an adhesive-type mechanism,^42^ in which the crystallizing species instantaneously find sites to incorporate into the crystallites in the spherulite.

The AFM images obtained from the micro/nanostructures formed in the central areas of deposits were then studied. In the central areas of deposits, the out-of-plane lamellae were infrequently found, and the semicrystalline structures were mostly in the form of in-plane terraces and spirals (see Figure 5). The terrace structure consists of in-plane layers which are located on top of each other, and each layer is made of amorphous and crystalline lamellae. The first layer, which was located directly over the glass substrate, was approximately twice thinner than other layers on top of the first layer (see Figure 5, the second column). The formation of such thick layers on top of the first thin monolayer has previously been observed for PEO homopolymers and copolymers containing PEO blocks.^55^ It is owing to the wetting behaviour of PEO, where the strong affinity of PEO with the hydrophilic substrate and the formation of a crystallized thin monolayer on top of the substrate create this interfacial interaction-induced lamellar ordering in the terrace.^55^ The growth mechanism of the islandlike two-dimensional layers of terraces is described as the two-dimensional nucleation growth (2DNG) mechanism,^42^ which is associated with diffusion-limited crystallization. The spiral structure consists of a layer which grows along a curve, which turns around a fixed center point originated from the outcrop of a screw dislocation,^56^ at an increasing distance from the base. The height of the layer was shown to gradually decrease from the base toward the vertex of the spiral (see Figure 5, the fourth column), which has previously been shown to be due to the slight tilt of the layer during the crystal growth.^22^ Such a mechanism of the crystal growth is described as the spiral growth mechanism.^42^ In the central areas of deposits, the tendency to form stacks of out-of-plane lamellae was observed to reduce even further as the initial concentration of solutions increased, while the tendency to form spirals increased (see Figure 5).

**Figure 10:**
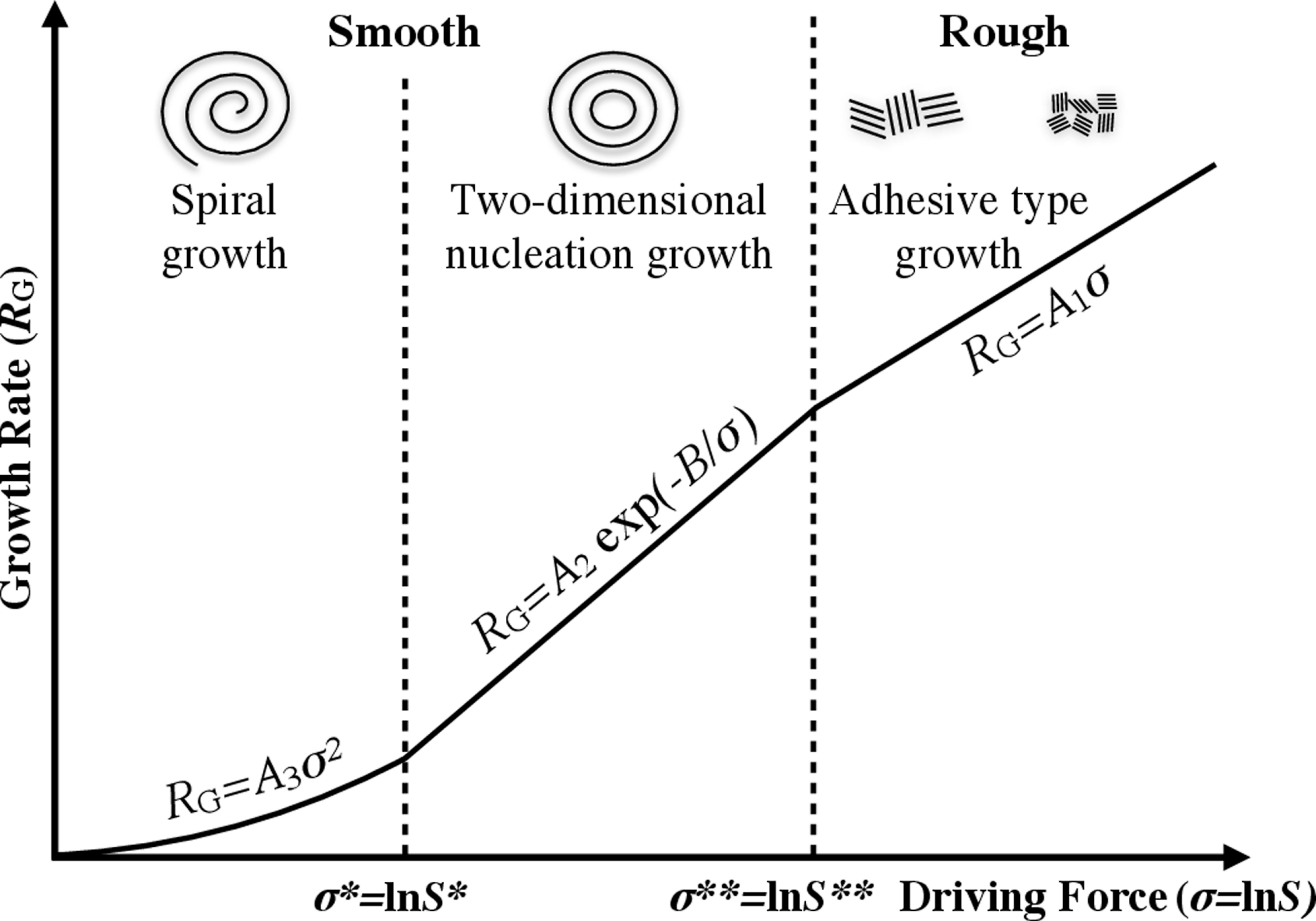
Schematic illustration of a crystal structural change in relation to the structure of interface, the growth mechanism, and the growth rate (*R*_G_) vs the crystallization driving force (σ), expressed as σ = ln *S* in which *S* is the ratio of supersaturation defined as . The critical driving forces of σ* and σ** are, respectively, expressed as σ* = ln S* and σ** = ln S** in which S* and S** are the critical ratios of supersaturation. The structure of the interface is classified as either rough or smooth. The relation between *R*_G_ and σ is different for each growth mechanism, including *R*_G_ = *A*_1_σ for the adhesive-type, *R*_G_ = *A*_2_ exp(−*B*/σ) for the 2DNG, and *R*_G_ = *A*_3_σ^2^ for the spiral growth mechanism, where *A*_1_, *A*_2_, *A*_3_, and *B* are constant values.^42^

The theoretical model developed in this study was designed to predict possible micro/nanostructures formed in different areas of the deposits based on the driving force of crystallization provided at that area of the droplets. The types of micro/nanostructures formed in different areas of the deposits were predicted by estimating the rate of supersaturation development at each area of the droplets. The rate of supersaturation development, which affects the micro/nanostructures of the resulting deposits through affecting the driving force of crystallization,^29,30,42^ was estimated by calculating the concentration change per unit of time at the saturation point, d*c*/d*t*|_*c*=*c*_sat__, for all droplets radii. The value of d*c*/d*t*|_*c*=*c*_sat__ was found to have an upward trend from the center to periphery of the solution droplets with four initial concentrations, while this value was lower for the droplets containing higher initial concentration solutions in each radius (see Figure 9). Figure 10 shows how the crystal structure is different depending on the atomic structure of the solid–liquid interface, the growth mechanism, and the growth rate versus the driving force of crystallization.^30,42^ Depending on the driving force of crystallization, three types of growth mechanisms are expected: (1) adhesive-type growth mechanism on rough interfaces, (2) 2DNG mechanism, and (3) spiral growth mechanisms on smooth interfaces. The relation between the growth rate (*R*_G_) and the crystallization driving force (σ) is different for each growth mechanism, including (1) *R*_G_ = *A*_1_σ for the adhesive-type, (2) *R*_G_ = *A*_2_ exp(−*B*/σ) for the 2DNG, and (3) *R*_G_ = *A*_3_σ^2^ for the spiral growth mechanism, where *A*_1_, *A*_2_, *A*_3_, and *B* are constant values. σ is expressed in a general form as σ = Δμ/*kT*, where *k* is the Boltzmann constant, *T* is the temperature, and Δμ is the difference in chemical potential, defined as Δμ = *kT* ln *S*, in which *S* is the ratio of supersaturation. σ is thus expressed as σ = ln *S*. Therefore, the driving force and supersaturation are directly correlated due to monotonically increasing property of the natural logarithm. The critical driving forces of σ* and σ** are obtained from where the graphs cross. Crystal structures with rough surfaces, for example, spherulites, are expected to form where the driving force is above σ** (see Figure 10), whereas crystals with smooth surfaces, for example, terraces and spirals, are expected to form where the driving force is below σ** (see Figure 10). Formation of spirals requires a lower driving force, that is, below σ*, compared to terraces which need a driving force between σ* and σ**.

The key comparisons between the numerical results obtained from the model and the experimental results obtained from the AFM study are outlined in Table 1. The table compares the numerical and experimental results for different areas in each deposit, that is, *r* < 0.5*R* within the central area and *r* > 0.95*R* within the periphery area (top two rows), and for different initial concentrations of solutions, which are categorized in two groups here: low initial concentrations, that is, 0.01 and 0.1% (w/w), and high initial concentrations, that is, 1 and 10% (w/w) (bottom two rows). A similar trend as shown in Figure 10 was apparent in the experimental and numerical results obtained from this study. The numerical results showed the droplet periphery had the highest level of d*c*/d*t*|_*c*=*c*_sat__, compared to all other radii, in each droplet (see Figure 9). The driving force of crystallization in the periphery area was thus higher than in the central area of each droplet. As a result of the high driving force of crystallization in the periphery, a large number of nuclei were formed and grew rapidly with the adhesive-type mechanism which created the stacks of out-of-plane lamellae in the periphery areas as observed using AFM (see Figure 4), whereas the low driving force condition occurred in the central areas of droplets, where a very small number of nuclei were typically formed and grew slowly with the 2DNG or spiral growth mechanisms; these growth mechanisms, respectively, caused the formation of terrace and spiral structures in the central areas of droplets as observed using AFM (see Figure 5). The spiral structures, which require a lower driving force to be formed (see Figure 10), were predominantly found in the central areas of the droplets containing higher initial concentration solutions (see Figure 5, the third and fourth columns). The solutions with higher initial concentrations were shown to have lower values of d*c*/d*t*|_*c*=*c*_sat__ in each radius compared to the solutions with lower initial concentrations (see Figure 9). Therefore, the numerical results agreed with and elucidated the experimental findings qualitatively, which are all summarized in Table 1.

**Table 1:**
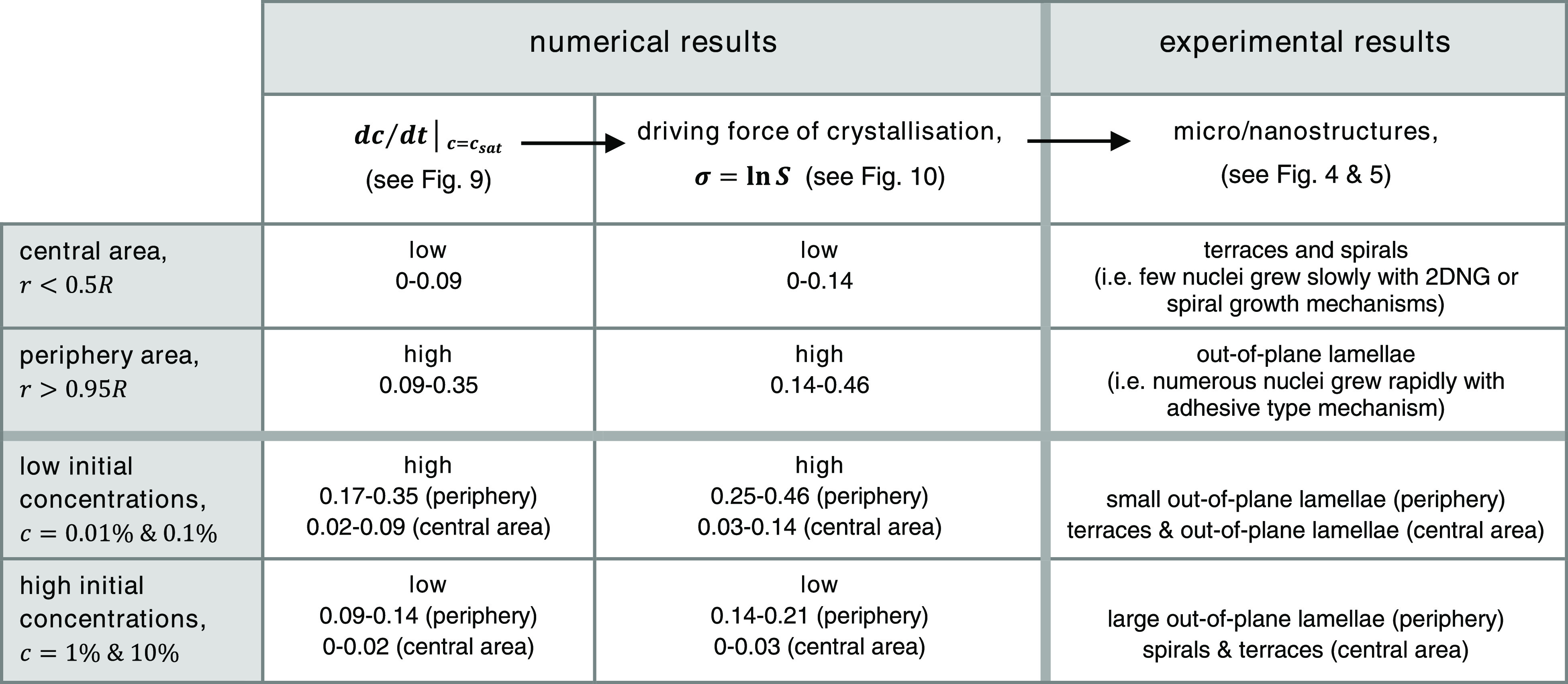
Brief Comparison of the Numerical and Experimental Results for Different Areas in Each Deposit, that is, *r* < 0.5*R* Within the Central Area and *r* > 0.95*R* Within the Periphery Area (Top Two Rows) and for Different Initial Concentrations of Solutions, Which are Categorized in Two Groups here: Low Initial Concentrations, that is, 0.01 and 0.1% (w/w), and High Initial Concentrations, that is, 1 and 10% (w/w) (Bottom Two Rows)^a^

a The data for d*c*/d*t*|_*c*=*c*_sat__ comes from Figure 9. The driving force of crystallization, σ = ln *S*, (see Figure 10) is calculated in the normalized unit of time. The resulting micro/nanostructures are described based on the AFM images shown in Figures 4 and 5.

Moreover, the experimental results for the conical pillar were compared with the numerical results. The value of d*c*/d*t*|_*c*=*c*_sat__ and thus the driving force of crystallization nearly approached zero in the center of the droplet containing the highest initial concentration solution, that is, 10% (w/w) solution (see Figure 9). In that area, a tall pillar was observed to form in the experiment (see Figure 3d,h), and the polymer micro/nanostructures formed over the pillar were observed to be predominantly amorphous and the semicrystalline structures were rarely found (see Figure 6). The formation of the amorphous structures could be the result of the nearly zero driving force of crystallization in that area (see Figure 9). The formation of the tall central pillar could have a relation to the difference between the density of amorphous polymer structures formed in the pillar and the semicrystalline polymer structures formed in the periphery and central areas. The polymer molecules in a crystalline structure are packed together more tightly and efficiently than in an amorphous structure, and thus the density of a semicrystalline structure is typically higher than that of the corresponding amorphous structure.^57^

The AFM analysis showed that the size of the lamellae varied for the deposits of different initial concentration solutions, which were in good agreement with the numerical results. According to the kinetics of crystallization in solution, the rate of supersaturation development in the solution is a crucial factor which alters the size of crystals.^29^ The nucleation rate is practically zero below a certain critical rate of supersaturation development; however, upon reaching this critical supersaturation rate, the nucleation can be initiated. At low supersaturation rates, crystals can grow faster than they nucleate, resulting in the formation of a small number of large crystals, whereas at higher supersaturation rates, the nucleation dominates the growth, resulting in the formation of a larger number of smaller crystals. The supersaturation rate, estimated as d*c*/d*t*|_*c*=*c*_sat__ in this study, thus controls the crystal size through affecting the competition between the nucleation- and growth-dominant crystallizations. The higher values of d*c*/d*t*|_*c*=*c*_sat__ in the periphery areas of the droplets containing lower initial concentration solutions (see Figure 9) compared to higher initial concentration solutions can be the reason for the formation of layers with shorter lengths (see Figure 4) and lower average thickness (see Figure 7a) in those areas. It is reasonable to expect some deviations in the layer thickness data which are reported as error bars in Figure 7a. Most errors are related to the cases where the chosen out-of-plane lamellae were slightly tilted; thus the values of the valley-to-valley intervals in the related phase profiles would slightly underestimate the true thicknesses. As each layer consists of amorphous and crystalline lamellae, the measured values reported in Figure 7a give the average thickness of the crystalline plus amorphous lamellae.^58^

The experimental and numerical results for the macrostructure of deposits were compared. The experimental results exhibited that the macrostructure of deposits changed from a ring to a tall central pillar formed over a puddle as the initial concentration of solutions increased from 0.01 to 10% (w/w) (see Figure 3). The numerical results showed that the macrostructure of deposits changed from a ring to a puddle as the initial concentration of solutions increased from 0.01 to 10% (w/w) (see Figure 8). The numerical results were in agreement with the experimental findings, with the exception that the stick-slip behavior,^51,52^ observed for the 0.1% solution, and the pillar formation, observed for the 10% solution, were not demonstrated by the model. The stick-slip behavior has previously been described^51,52,59−61^ using the theoretical approaches based on the Young’s equation which defines the equilibrium contact angle at the triple contact line of droplet. The depinning of the contact line has been explained to occur as a result of the unbalanced Young's force at the contact line, which then jumps to its next equilibrium position. The excess Gibbs free energy before the jump of the contact line to a more thermodynamically favorable position has been calculated for each stick-slip motion of the droplet.^59−61^ The pillar formation has also been shown previously to be affected by the Peclet number, that is, the ratio of the advective to diffusive mass transfer, within the evaporating solution droplet.^8,10,11^ The high Peclet numbers (Pe ≫ 1) have been associated with the ring and pillar formations in the deposits of the droplets containing low-molecular-weight PEO solutions (<300 kg/mol) as the diffusion is not sufficient to homogenize the solute concentration during evaporation.^8^ For higher molecular weight PEO solutions (>300 kg/mol), however, the interfacial interaction phenomena have been shown to cause the pillar formation to suppress and a puddlelike deposit to form.^7^ The interfacial interaction phenomena are due to the intertwining between the long loops and tails of the polymer molecules adsorbed on the substrate and the polymer gel network formed inside the droplet as water evaporates.^7^ Accordingly, the stick-slip behavior and the pillar formation, observed in the experimental findings (see Figure 3), are usually hard to be modeled using a complete fluid dynamical system with limited boundary condition information and is beyond the scope of this investigation.

## Conclusions

In summary, this work presents an experimental and mathematical approach to study the macrostructures and micro/nanostructures of deposits formed after droplet evaporation of solutions containing crystallizable solutes. The influence of initial solution concentration on the resulting deposits after evaporation of droplets containing aqueous PEO solutions were studied using stereo microscopy and AFM. A model based on thin-film lubrication theory was also presented and numerically solved using finite difference methods. The final heights of droplets, , were calculated to describe the macrostructure of deposits. Then, the concentration changes per unit of time on the saturation point, d*c*/d*t*|_*c*=*c*_sat__, were calculated to estimate the rates of supersaturation development which affect the micro/nanostructures formed at each area of deposits considering the kinetics of crystallization. The AFM study revealed that the deposits predominantly consisted of semicrystalline micro/nanostructures in the form of out-of-plane lamellae at the periphery areas and in the form of in-plane terraces or spirals in the central areas. As the initial concentration of solutions increased, the tendency to form larger out-of-plane lamellae increased at the periphery areas and the tendency to form spirals increased in the central areas of deposits. The formation of these micro/nanostructures was qualitatively justified by the model as a similar trend was apparent in the numerical results; the level of d*c*/d*t*|_*c*=*c*_sat__ and thus the driving force of crystallization had an upward trend from the center to periphery of all droplets, while the solutions with higher initial concentrations had lower levels of d*c*/d*t*|_*c*=*c*_sat__ in each radius. The higher driving forces of crystallization in the periphery areas of droplets formed the rough surfaces of out-of-plane lamellae, while the lower driving forces in the central areas of droplets formed the smooth surfaces of in-plane terraces and spirals. Also, the lower driving forces of crystallization in the higher initial concentration solutions caused the formation of larger out-of-plane lamellae at the periphery areas and spirals in the central areas of the deposits. Therefore, the theoretical results showed a good agreement with the experimental findings. Consequently, this study presents important experimental findings about the micro/nanostructures of PEO deposits linked with numerical results that complement the findings about the macrostructure of deposits for various potential engineering and biochemical applications.

## Supporting Information Available

Detailed derivation of the formulation to find the droplet height and the concentration change per unit of time on the saturation boundary (PDF)
