# Supplementary material for: Semicrystalline Polymer Micro/Nanostructures Formed by Droplet Evaporation of Aqueous Poly(ethylene oxide) Solutions: Effect of Solution Concentration
Source: Langmuir. 2022 Nov 28;38(49):15063–76. doi: 10.1021/acs.langmuir.2c01872 (PMC9753751; doi:10.1021/acs.langmuir.2c01872)
Supplement: Supplementary file 1 — la2c01872_si_001.pdf [file la2c01872_si_001.pdf]

Supporting Information

Semicrystalline Polymer Micro/Nanostructures Formed by Droplet Evaporation of Aqueous Poly(ethylene oxide) (PEO) solutions: Effect of Solution Concentration

Shadi Kolahgar-Azari,[†] Antonia Kagkoura,[†] Dimitrios Mamalis,[‡] Jane R. Blackford,[†] Prashant Valluri,[§] Khellil Sefiane,[§] and Vasileios Koutsos[†]*

[†]School of Engineering, Institute for Materials and Processes, The University of Edinburgh, King's Buildings, Edinburgh EH9 3FB, United Kingdom

[‡]Offshore Renewable Energy Catapult, Offshore House, Albert Street, Blyth, NE24 1LZ, United Kingdom

[§]School of Engineering, Institute for Multiscale Thermofluids, The University of Edinburgh, King's Buildings, Edinburgh EH9 3FD, United Kingdom

[*]Corresponding Author Email: vasileios.koutsos@ed.ac.uk. Tel.: +44 (0) 131 6508704. Fax: +44 (0) 131 6506554.

Droplet Height, $\boldsymbol{h(r,t)}$

The droplet height was obtained by numerically solving the equations 2 and 9. After discretization of the equations, a difference equation was obtained which was then represented by a sparse matrix equation. The detailed derivations of the formulations are described in the following.

To obtain the discretized $h(r,t)$ from equation 2, the discretized $v(r,t)$ needed to be firstly obtained from equation 9, which consists of three derivations. The three derivations were carried out, respectively, as described in the following.

The first derivation was

$$\frac{\partial h(r,t)}{\partial r} = \lim_{\Delta r \to 0} \frac{h(r+\Delta r,t) - h(r,t)}{\Delta r} \simeq \frac{h(r+\Delta r,t) - h(r,t)}{\Delta r}, \qquad (1)$$

in which the limit was ignored by selecting very small values of Δr. The second derivation was

$$\frac{\partial}{\partial r}\left(r\frac{\partial h(r,t)}{\partial r}\right) = \frac{\partial}{\partial r}\left(\frac{rh(r+\Delta r,t) - rh(r,t)}{\Delta r}\right)$$

$$= \frac{1}{\Delta r^2}[(r+\Delta r)h(r+2\Delta r,t) - (r+\Delta r)h(r+\Delta r,t)$$

$$-rh(r+\Delta r,t) + rh(r,t)]. \qquad \text{(S2)}$$

The third one constituted

$$\frac{\partial}{\partial r}\left(\frac{1}{r}\frac{\partial}{\partial r}\left(r\frac{\partial h(r,t)}{\partial r}\right)\right) =$$

$$\frac{\partial}{\partial r}\left(\frac{1}{\Delta r^2}\left[\left(\frac{r+\Delta r}{r}\right)h(r+2\Delta r,t) - \left(\frac{r+\Delta r}{r}\right)h(r+\Delta r,t)\right.\right.$$

$$\left.\left. - h(r+\Delta r,t) + h(r,t)\right]\right) =$$

$$\frac{1}{\Delta r^3}\left[\left(\frac{r+2\Delta r}{r+\Delta r}\right)h(r+3\Delta r,t) - \left(\frac{r+2\Delta r}{r+\Delta r}\right)h(r+2\Delta r,t)\right.$$

$$- h(r+2\Delta r,t) + h(r+\Delta r,t)$$

$$- \left(\frac{r+\Delta r}{r}\right)h(r+2\Delta r,t) + \left(\frac{r+\Delta r}{r}\right)h(r+\Delta r,t)$$

$$\left. + h(r+\Delta r,t) - h(r,t)\right] = \qquad \text{(S3)}$$

$$\frac{1}{\Delta r^3}\left[\left(\frac{r+2\Delta r}{r+\Delta r}\right)h(r+3\Delta r,t)-\left(\frac{r+2\Delta r}{r+\Delta r}+1+\frac{r+\Delta r}{r}\right)h(r+2\Delta r,t)\right.$$
$$\left.+\left(2+\frac{r+\Delta r}{r}\right)h(r+\Delta r,t)-h(r,t)\right],$$

which was defined as $g(r,t)$ here. The discretized $v(r,t)$ was thus described as

$$v(r,t)=\frac{h(r,t)^2\gamma}{3\mu}g(r,t)\,, \tag{S4}$$

in which μ was inserted from equation 10. The discretized $v(r,t)$ thus constituted

$$v(r,t)=\frac{h(r,t)^2\gamma}{3\mu_0}\,e^{-a(\frac{c(r,t)}{c_{sat}})^b}g(r,t)\,. \tag{S5}$$

By introducing it to equation 2, the discretized $h(r,t)$ was found. For that, $\partial(rh(r,t)\,v(r,t))/\partial r$ was written as

$$\begin{aligned}\frac{\partial\big(rh(r,t)v(r,t)\big)}{\partial r}&=\frac{\partial\left(rh(r,t)\times\dfrac{h(r,t)^2\gamma}{3\mu_0}\,e^{-a(\frac{c(r,t)}{c_{sat}})^b}g(r,t)\right)}{\partial r}\\&=\frac{\gamma}{3}\frac{\partial\left(\dfrac{rh(r,t)^3}{\mu_0}e^{-a(\frac{c(r,t)}{c_{sat}})^b}g(r,t)\right)}{\partial r}\\&=\frac{\gamma}{3}\frac{\partial\big(r\,f(r,t)\,g(r,t)\big)}{\partial r},\end{aligned} \tag{S6}$$

where $f(r,t)$ was defined as

$$f(r,t)=\frac{h(r,t)^3}{\mu_0}e^{-a(\frac{c(r,t)}{c_{sat}})^b}, \tag{S7}$$

and $\bar{g}(r,t)$ was defined as

$$\bar{g}(r,t) = rg(r,t)\,. \tag{S8}$$

Therefore, $\partial(rh(r,t)\ v(r,t))/\partial r$ was written as

$$\begin{aligned}\frac{\partial\big(rh(r,t)v(r,t)\big)}{\partial r} &= \frac{\gamma}{3}\frac{\partial\big(f(r,t)\bar{g}(r,t)\big)}{\partial r} \\ &= \frac{\gamma}{3}\frac{1}{\Delta r}\big(f(r+\Delta r,t)\bar{g}(r+\Delta r,t) - f(r,t)\bar{g}(r,t)\big)\,,\end{aligned} \tag{S9}$$

in which $f(r,t)$ and $f(r+\Delta r,t)$ were known, and $\bar{g}(r,t+\Delta t)$ and $\bar{g}(r+\Delta r,t+\Delta t)$, defined earlier, were substituted as

$$\begin{aligned}&\frac{\partial\big(rh(r,t)v(r,t)\big)}{\partial r} \\ &\quad= \frac{\gamma}{3}\frac{1}{\Delta r}f(r+\Delta r,t) \\ &\quad\times\frac{1}{\Delta r^3}\Bigg[(r+\Delta r)\left(\frac{r+3\Delta r}{r+2\Delta r}\right)h(r+4\Delta r,t) - (r \\ &\quad+\Delta r)\left(\frac{r+3\Delta r}{r+2\Delta r}+1+\frac{r+2\Delta r}{r+\Delta r}\right)h(r+3\Delta r,t)+(r \\ &\quad+\Delta r)\left(2+\frac{r+2\Delta r}{r+\Delta r}\right)h(r+2\Delta r,t)-(r+\Delta r)h(r+\Delta r,t)\Bigg] \\ &\quad-\frac{\gamma}{3}\frac{1}{\Delta r}f(r,t) \\ &\quad\times\frac{1}{\Delta r^3}\Bigg[r\left(\frac{r+2\Delta r}{r+\Delta r}\right)h(r+3\Delta r,t) \\ &\quad-r\left(\frac{r+2\Delta r}{r+\Delta r}+1+\frac{r+\Delta r}{r}\right)h(r+2\Delta r,t) \\ &\quad+r\left(2+\frac{r+\Delta r}{r}\right)h(r+\Delta r,t)-rh(r,t)\Bigg]\end{aligned} \tag{S10}$$

It then was written as

$$\frac{1}{r}\frac{\partial\big(rh(r,t)v(r,t)\big)}{\partial r}$$

$$= a_4 h(r+4\Delta r,t) + a_3 h(r+3\Delta r,t) + a_2 h(r+2\Delta r,t) + a_1 h(r+\Delta r,t) + a_0 h(r,t)\ , \tag{S11}$$

in which a_4, a_3, a_2, a_1, and a_0 were defined as

$$a_4 = \frac{\gamma}{3}\frac{1}{\Delta r^4} f(r+\Delta r,t)\left(\frac{r+\Delta r}{r}\right)\left(\frac{r+3\Delta r}{r+2\Delta r}\right), \tag{S12}$$

$$a_3 = -\frac{\gamma}{3}\frac{1}{\Delta r^4}\left(f(r+\Delta r,t)\left(\frac{(r+\Delta r)(r+3\Delta r)}{r(r+2\Delta r)} + \frac{2r+3\Delta r}{r}\right) + f(r,t)\left(\frac{r+2\Delta r}{r+\Delta r}\right)\right), \tag{S13}$$

$$a_2 = \frac{\gamma}{3}\frac{1}{\Delta r^4}\left(f(r+\Delta r,t)\left(\frac{3r+4\Delta r}{r}\right) + f(r,t)\left(\frac{r+2\Delta r}{r+\Delta r} + \frac{2r+\Delta r}{r}\right)\right), \tag{S14}$$

$$a_1 = -\frac{\gamma}{3}\frac{1}{\Delta r^4}\left(\left(\frac{r+\Delta r}{r}\right) f(r+\Delta r,t) + f(r,t)\left(\frac{3r+\Delta r}{r}\right)\right), \tag{S15}$$

$$a_0 = \frac{\gamma}{3}\frac{1}{\Delta r^4} f(r,t)\ . \tag{S16}$$

After inserting the discretized $\partial(rh(r,t)\ v(r,t))/r\partial r$ in equation 2, the equation was written as

$$\frac{h(r,t+\Delta t) - h(r,t)}{\Delta t} \tag{S17}$$

$$= -\big(a_4 h(r + 4\Delta r, t) + a_3 h(r + 3\Delta r, t) + a_2 h(r + 2\Delta r, t)$$
$$+ a_1 h(r + \Delta r, t) + a_0 h(r, t)\big) - J(r, t).$$

Moving the unknown parameter to the left-hand side and all the knowns to the right-hand side, the equation constituted

$$\begin{aligned} h(r, t + \Delta t) = &-\Delta t\big(a_4 h(r + 4\Delta r, t) + a_3 h(r + 3\Delta r, t) \\ &+ a_2 h(r + 2\Delta r, t) + a_1 h(r + \Delta r, t) + a_0 h(r, t)\big) \\ &- \Delta t J(r, t) + h(r, t), \end{aligned} \tag{S18}$$

which was then written as

$$\begin{aligned} h(r, t + \Delta t) = &\big((1 - a_0 \Delta t) h(r, t) \\ &- a_1 \Delta t h(r + \Delta r, t) - a_2 \Delta t h(r + 2\Delta r, t) \\ &- a_3 \Delta t h(r + 3\Delta r, t) - a_4 \Delta t h(r + 4\Delta r, t)\big) - \Delta t J(r, t). \end{aligned} \tag{S19}$$

Here, r, $h(r, t + \Delta t)$, $h(r, t)$ and $J(r, t)$ were introduced as the vectors $\vec{r}$, $\vec{h}(t + \Delta t)$, $\vec{h}(t)$ and $\vec{J}(t)$, shown in the column matrices below;

$$\vec{r} = \begin{bmatrix} 0 \\ \Delta r \\ 2\Delta r \\ \vdots \\ R - \Delta r \\ R \end{bmatrix} \qquad \vec{h}(t + \Delta t) = \begin{bmatrix} h(0, t + \Delta t) \\ h(\Delta r, t + \Delta t) \\ h(2\Delta r, t + \Delta t) \\ \vdots \\ h(R - \Delta r, t + \Delta t) \\ h(R, t + \Delta t) \end{bmatrix} \tag{S20}$$

$$\vec{h}(t) = \begin{bmatrix} h(0,t) \\ h(\Delta r,t) \\ h(2\Delta r,t) \\ \vdots \\ h(R-\Delta r,t) \\ h(R,t) \end{bmatrix} \qquad \vec{J}(t) = \begin{bmatrix} J(0,t) \\ J(\Delta r,t) \\ J(2\Delta r,t) \\ \vdots \\ J(R-\Delta r,t) \\ J(R,t) \end{bmatrix}.$$

The sparse matrix M_1 was also introduced as

$$M_1 = \begin{bmatrix} 1-a_0\Delta t & -a_1\Delta t & -a_2\Delta t & -a_3\Delta t & -a_4\Delta t & 0 & \cdots & \cdots & \cdots & 0 \\ 0 & 1-a_0\Delta t & -a_1\Delta t & -a_2\Delta t & -a_3\Delta t & -a_4\Delta t & 0 & \ddots & \ddots & \vdots \\ 0 & 0 & 1-a_0\Delta t & -a_1\Delta t & -a_2\Delta t & -a_3\Delta t & -a_4\Delta t & \ddots & \ddots & 0 \\ \vdots & \ddots & \ddots & \ddots & \ddots & \ddots & \ddots & \ddots & 1-a_0\Delta t & -a_1\Delta t \\ 0 & \cdots & \cdots & \cdots & \cdots & \cdots & \cdots & \cdots & 0 & 1-a_0\Delta t \end{bmatrix}. \tag{S21}$$

The sparse matrix operation to calculate $h(r,t+\Delta t)$ therefore constituted

$$\begin{bmatrix} h(0,t+\Delta t) \\ h(\Delta r,t+\Delta t) \\ h(2\Delta r,t+\Delta t) \\ \vdots \\ h(R-\Delta r,t+\Delta t) \\ h(R,t+\Delta t) \end{bmatrix} = \begin{bmatrix} 1-a_0\Delta t & -a_1\Delta t & -a_2\Delta t & -a_3\Delta t & -a_4\Delta t & 0 & \cdots & \cdots & \cdots & 0 \\ 0 & 1-a_0\Delta t & -a_1\Delta t & -a_2\Delta t & -a_3\Delta t & -a_4\Delta t & 0 & \ddots & \ddots & \vdots \\ 0 & 0 & 1-a_0\Delta t & -a_1\Delta t & -a_2\Delta t & -a_3\Delta t & -a_4\Delta t & \ddots & \ddots & 0 \\ \vdots & \ddots & \ddots & \ddots & \ddots & \ddots & \ddots & \ddots & 1-a_0\Delta t & -a_1\Delta t \\ 0 & \cdots & \cdots & \cdots & \cdots & \cdots & \cdots & \cdots & 0 & 1-a_0\Delta t \end{bmatrix} \cdot \begin{bmatrix} h(0,t) \\ h(\Delta r,t) \\ h(2\Delta r,t) \\ \vdots \\ h(R-\Delta r,t) \\ h(R,t) \end{bmatrix} - \Delta t \begin{bmatrix} J(0,t) \\ J(\Delta r,t) \\ J(2\Delta r,t) \\ \vdots \\ J(R-\Delta r,t) \\ J(R,t) \end{bmatrix}, \tag{S22}$$

which was briefly written as

$$h(r,t+\Delta t) = M_1 \cdot h(r,t) - \Delta t\, J(r,t). \tag{S23}$$

Solute Concentration, $c(r,t)$

The solute mass, $c(r,t)h(r,t)$, which is short noted by $ch(r,t)$ here, and then the solute concentration, $c(r,t)$, were obtained by numerically solving the equations 4 and 9. For that, the discretization of the equations was carried out, and then, the resulting difference equation was turned into a sparse matrix equation as described in the following.

To discretize $ch(r,t)$ based on equation 4, the discretized $v(r,t)$ was firstly substituted from equation S5 to obtain $\partial(rch(r,t)\,v(r,t))/\partial r$ as

$$\frac{\partial\big(rch(r,t)v(r,t)\big)}{\partial r}=\frac{\partial\left(rch(r,t)\times\frac{h(r,t)^2\gamma}{3\mu_0}\,e^{-a(\frac{c(r,t)}{c_{sat}})^b}g(r,t)\right)}{\partial r}$$

$$=\frac{\gamma}{3}\frac{\partial\left(ch(r,t)\times\frac{rh(r,t)^2}{\mu_0}e^{-a(\frac{c(r,t)}{c_{sat}})^b}\times g(r,t)\right)}{\partial r}$$

$$=\frac{\gamma}{3}\frac{\partial\big(ch(r,t)\times l(r,t)\times g(r,t)\big)}{\partial r}, \qquad \text{(S24)}$$

where $l(r,t)$ was defined as

$$l(r,t)=\frac{rh(r,t)^2}{\mu_0}e^{-a(\frac{c(r,t)}{c_{sat}})^b}. \qquad \text{(S25)}$$

Then, $\partial(rch(r,t)\,v(r,t))/\partial r$ was written as

$$\frac{\partial\big(rch(r,t)v(r,t)\big)}{\partial r}=$$

$$=\frac{\gamma}{3}\frac{1}{\Delta r}\big(ch(r+\Delta r,t)l(r+\Delta r,t)g(r+\Delta r,t) \qquad \text{(S26)}$$

$$-ch(r,t)l(r,t)g(r,t)\big).$$

After inserting the discretized $\partial(rch(r,t)\ v(r,t))/\partial r$ in equation 4, the equation was written as

$$ch(r,t+\Delta t) =$$

$$ch(r,t) - \frac{\gamma}{3r}\frac{\Delta t}{\Delta r}\big(ch(r+\Delta r,t)l(r+\Delta r,t)g(r+\Delta r,t) - ch(r,t)l(r,t)g(r,t)\big). \quad \text{(S27)}$$

The equation was then written as

$$ch(r,t+\Delta t) = b_0(r)ch(r,t) + b_1(r+\Delta r)ch(r+\Delta r,t), \quad \text{(S28)}$$

where

$$b_0(r) = 1 + \frac{\gamma}{3r}\frac{\Delta t}{\Delta r}l(r,t)g(r,t), \quad \text{(S29)}$$

$$b_1(r+\Delta r) = -\frac{\gamma}{3r}\frac{\Delta t}{\Delta r}l(r+\Delta r,t)g(r+\Delta r,t). \quad \text{(S30)}$$

$ch(r,t+\Delta t)$ is a short note of $c(r,t+\Delta t)h(r,t+\Delta t)$, and $ch(r,t)$ is a short note of $c(r,t)h(r,t)$. Here, $h(r,t+\Delta t)$ and $h(r,t)$ were considered as the vectors $\vec{h}(t+\Delta t)$ and $\vec{h}(t)$, described in S20, and $c(r,t+\Delta t)$ and $c(r,t)$ were introduced as the vectors $\vec{c}(t+\Delta t)$ and $\vec{c}(t)$, described below;

$$\vec{c}(t+\Delta t) = \begin{bmatrix} c(0,t+\Delta t) \\ c(\Delta r,t+\Delta t) \\ c(2\Delta r,t+\Delta t) \\ \vdots \\ c(R-\Delta r,t+\Delta t) \\ c(R,t+\Delta t) \end{bmatrix}, \qquad \vec{c}(t) = \begin{bmatrix} c(0,t) \\ c(\Delta r,t) \\ c(2\Delta r,t) \\ \vdots \\ c(R-\Delta r,t) \\ c(R,t) \end{bmatrix}. \quad \text{(S31)}$$

The sparse matrix M_2 was also introduced as

$$M_2 = \begin{bmatrix} b_0(0) & b_1(\Delta r) & 0 & 0 & 0 & \cdots & 0 \\ 0 & b_0(\Delta r) & b_1(2\Delta r) & 0 & 0 & \ddots & \vdots \\ 0 & 0 & b_0(2\Delta r) & b_1(3\Delta r) & 0 & \cdots & \vdots \\ \vdots & \ddots & \ddots & \ddots & \ddots & b_0(R-\Delta r) & b_1(R) \\ 0 & 0 & 0 & \cdots & \cdots & 0 & b_0(R) \end{bmatrix}, \tag{S32}$$

The sparse matrix operation to calculate $ch(r, t+\Delta t)$ thus constituted

$$\begin{bmatrix} ch(0, t+\Delta t) \\ ch(\Delta r, t+\Delta t) \\ ch(2\Delta r, t+\Delta t) \\ \vdots \\ ch(R-\Delta r, t+\Delta t) \\ ch(R, t+\Delta t) \end{bmatrix} = \begin{bmatrix} b_0(0) & b_1(\Delta r) & 0 & 0 & 0 & \cdots & 0 \\ 0 & b_0(\Delta r) & b_1(2\Delta r) & 0 & 0 & \ddots & \vdots \\ 0 & 0 & b_0(2\Delta r) & b_1(3\Delta r) & 0 & \cdots & \vdots \\ \vdots & \ddots & \ddots & \ddots & \ddots & b_0(R-\Delta r) & b_1(R) \\ 0 & 0 & 0 & \cdots & \cdots & 0 & b_0(R) \end{bmatrix} \cdot \begin{bmatrix} ch(0, t) \\ ch(\Delta r, t) \\ ch(2\Delta r, t) \\ \vdots \\ ch(R-\Delta r, t) \\ ch(R, t) \end{bmatrix}, \tag{S33}$$

which was briefly written as

$$ch(r, t+\Delta t) = M_2 \cdot ch(r, t). \tag{S34}$$

$c(r, t+\Delta t)$ was then calculated by

$$c(r, t+\Delta t) = ch(r, t+\Delta t)/h(r, t+\Delta t). \tag{S35}$$

For the points $h(r, t + \Delta t)$ is zero, i.e. droplet periphery, $c(r, t + \Delta t)$ was extrapolated using neighboring points. Accordingly, the concentration changes per unit of time on the saturation boundary, $dc/dt\,\big|_{c=c_{sat}}$, for different droplets radii were calculated.
